# “I just wish it becomes part of routine care”: healthcare providers’ knowledge, attitudes and perceptions of screening for maternal mental health during and after pregnancy: a qualitative study

**DOI:** 10.1186/s12888-019-2261-x

**Published:** 2019-09-10

**Authors:** Mary Mccauley, Brown Abigail, Ofosu Bernice, Nynke Van Den Broek

**Affiliations:** 1 Centre for Maternal and Newborn Health, Liverpool School of Tropical Medicine, Pembroke Place, Liverpool, L3 5QA UK; 2 Korle-Bu Teaching Hospital, Accra, Ghana

**Keywords:** Maternal mental health, Healthcare providers, Quality of care, Antenatal care, Postnatal care, Psychological ill-health

## Abstract

**Background:**

Maternal mental health is an international public health concern. Many women experience mental ill-health during and after pregnancy, but assessment is not part of routine maternity care in many low- and middle-income countries. Healthcare providers are in a position to identify and support women who experience mental health disorders during and after pregnancy. We sought to investigate the knowledge, attitudes and perceptions of routine screening for maternal mental health during and after pregnancy among healthcare providers providing routine maternity care in Accra, Ghana. Enabling factors, barriers and potential management options to routinely screen maternal mental health during and after pregnancy were explored.

**Methods:**

Semi-structured key informant interviews (*n* = 20) and one focus group discussion (*n* = 4) were conducted with healthcare providers working in one public hospital in Accra, Ghana. Transcribed interviews were coded by topic and then grouped into categories. Thematic framework analysis was undertaken to identify emerging themes.

**Results:**

Most healthcare providers are aware of the importance of maternal mental health and would be keen to help women who experience mental ill-health during and after pregnancy, if resources were available to do so. An enabling factor was the suggestion of introducing a culturally appropriate mental health screening tool. However, compromised mental health was often considered a ‘spiritual issue’ and not routinely screened for by healthcare providers, nor requested by women. Barriers to the provision of quality maternal mental health care included lack of trained staff and lack of time.

**Conclusions:**

Healthcare providers are aware of the problem of the lack of maternal mental health provision during and after pregnancy and are open to developing protocols to improve care. Currently, screening for maternal mental ill-health is not part of routine maternity care. The establishment of such a service requires the reprioritisation of workloads, further training, and a change in the attitudes and practices of healthcare providers. Education to change the attitudes of healthcare providers, women and the wider community towards mental health is needed. The development and implementation of culturally appropriate guidelines would be beneficial and result in better quality of maternity care.

## Background

Maternal mental health is an international public health concern [1]. Many women experience mental health disorders during and after pregnancy that are often not recognised or treated [1, 2]. Health has previously been described as ‘a state of complete (physical, psychological and social) well-being and not merely the absence of disease or infirmity’ [3]. The updated Sustainable Development Goal 3 (SDG) aims to improve the health and well-being for all at all ages by 2030, and the Global Strategy for Women’s, Children’s and Adolescent’s Health emphasises that all women have the right to, and should obtain, the highest attainable standard of health, including physical and psychological care [4, 5]. In many low- and middle-income countries (LMIC) maternal mental health has been ignored and not part of the overall routine wellbeing and assessment of women. As a component of health, there is a need to focus on maternal mental health [2].

Maternal mental health is defined as ‘a state of well-being in which a mother realises her own abilities, can cope with the normal stresses of life, can work productively and fruitfully and is able to contribute to her community’ [6]. Common mental disorders (depression, anxiety) rank third in the list of the burden of disease globally and are expected to rank number one by 2030, overtaking road accidents and heart disease [7]. Globally, mental health disorders affect up to 10% of women during pregnancy and 13% of women following childbirth [6]. In LMIC, these figures are estimated to be as high as 15.6% of women during pregnancy and 19.8% of women following childbirth [6]. However, maternal mental health is often not reported, infrequently recognised and under-treated in many LMIC [8]. It is estimated that 1 in 4 women in LMIC report antepartum depression and 1 in 5 report postpartum depression; twice the rate of women in high income countries [9]. Women in LMIC are more vulnerable to contributing risk factors such as low socioeconomic status, unplanned pregnancies, low social support and domestic violence, all of which can increase the likelihood of a woman developing a mental disorder during or after pregnancy [10, 11].

Compromised maternal mental health is associated with adverse consequences for the mother and the baby, both short and long term [12, 13]. Many high-income countries such as the United Kingdom (UK), recognise the detrimental impact of maternal mental health disorders on the overall well-being of mothers and their newborn babies during and after pregnancy, and have implemented routine screening of mental health during and after pregnancy by a trained healthcare provider [14].

In Ghana, it is estimated that 650,000 people suffer from a severe mental disorder, 2,166,000 suffer from a moderate to mild mental disorder; but the number of women suffering from maternal mental health disorders is unknown [15]. General mental health services in Ghana are provided through specialized psychiatric hospitals in the capital, with little government funding for general hospital and primary healthcare level services [15]. Ghana has recently developed a comprehensive Mental Health Bill which aims to protects the rights of people with mental disorders in accordance with international human rights standards [15].

There are international policies and guidelines to improve maternal mental health but, at present, there is little practical implementation across many LMIC, including Ghana [15, 16]. Globally, 85% of women attend for antenatal care (ANC) at least once and this provides an opportunity for healthcare providers to improve the quality of care women receive [17]. It is therefore essential that healthcare providers are enabled to provide comprehensive and holistic maternity care that goes beyond the provision of basic emergency care and includes mental health assessment and management during and after pregnancy [2, 4, 5].

This study sought to investigate the knowledge, attitudes and perceptions of healthcare providers who provide routine maternity care, regarding maternal mental health during and after pregnancy in Accra, Ghana. In addition, enabling factors and barriers to the provision of mental health assessment were explored and potential management options, and how to translate these recommendations into clinical practice were considered.

## Methods

### Study design and setting

Data collection used a qualitative descriptive approach, and semi-structured key informant interviews (KII) and a focus group discussion (FGD) were conducted with healthcare providers working in the obstetric department of the largest teaching hospital in Accra, Ghana in May and June 2017. All interviews were held in a private location (an office in the healthcare facility) that would ensure privacy and that was convenient for the participants.

### Participants

Healthcare providers (mainly doctors) were included if they provided routine maternity care in the chosen study site. All participants were aged 21 years or more. Nurse-midwives were included to enable the triangulation of the data and broadened the scope of the topic. Snowballing and opportunistic techniques were employed to identify the participants. Participants were chosen purposively, based on their ability to speak English, and were recruited sequentially until saturation was met.

### Topic guide

The topic guide was designed by the primary researcher (AB) and piloted by two Obstetricians who were based in the study site. The topic guide was a flexible tool that enabled the interviewer to capture the healthcare providers’ responses as well as acting as a cue to probe further to develop an understanding of the participants perceptions and beliefs. Following the pilot of the topic guide it was amended and refined; for example, information related to the type of information that was being collected was clarified so that participants were aware that their general views were being sought and not first-hand experiences of mental health disorders during or after pregnancy. In addition to sociodemographic questions, the topic guide included five main subject areas: (1) overall understanding of mental health; (2) cultural perception of mental health disorders; (3) current experience and views on maternal mental health; (4) current management for women who experience mental disorders; and (5) suggestions for change to provide maternal mental healthcare.

### Data collection

The data collection was undertaken by a female student undertaking a Master’s in International Public Health. The data collector had completed training in qualitative research methods and was supported during the interview process by a second researcher (BO). Participants were approached by the data collector (AB) face-to-face prior to the interview and were given written and verbal information about the study. The information included an overview of the research aims, objectives and questions. An appointment was then scheduled in a convenient place and time for each participant. All participants were interviewed in English and the interviews and the focus group discussion lasted on average 30 mins. Interviews and the focus group discussion were conducted face-to-face, recorded on a digital recording device and transcribed upon completion. Triangulation data from the nurse-midwives’ interviews established credibility. By emphasising anonymity and confidentiality we increased participants confidence in providing honest answers.

### Analysis

The interviews and focus group discussion were transcribed and manually coded by the first researcher (AB). The second reviewer (BO) independently coded 50% of all transcripts. The identified codes were grouped into categories and reviewed by three researchers (AB, BO, MMC) to ensure consistency and to check for interrater reliability. This enabled the first extraction of data [18]. Key themes were then discussed and checked by all three researchers together to reach consensus (AB, BO, MMC). This helped to remove potential bias and strengthen the results.

### Ethics

Ethical approval was granted by the Liverpool School of Tropical Medicine, UK (LSTM14.025) and by the Korle-Bu Teaching Hospital, Accra, Ghana (KBTH-STC 00055/2017). Written informed consent was obtained from all participants of the study.

## Results

### Participants’ characteristics

A total of 24 healthcare providers participated in the study (20 doctors and 4 nurse-midwives; of whom 13 were female and 11 male). Two other participants who were approached refused to take part due to time constraints. Twenty doctors with varying levels of experience (junior doctor, specialist registrar, consultant) were interviewed using KII and four nurse-midwives took part in one FGD. Participants were aged between 25 and 65 years of age, with the majority between 25 and 35 years. Most participants were junior doctors and had between one to five years of experience of providing routine maternity care. All participants were interviewed in English.

### Emerging themes

The main emerging themes derived from the data included (1) enabling factors, (2) barriers and challenges and (3) healthcare providers’ suggestions on improving maternal mental health assessment. The variation in type of interview, age, gender or cadre of healthcare provider did not impact the response patterns.

### Enablers

Key enabling themes to improve the quality of routine maternity care included healthcare providers’ awareness of mental health in general and the understanding that women were at high risk of mental health disorders during and after pregnancy. Most healthcare providers expressed awareness that mental health covered a spectrum of disorders varying in severity (Table 1: Q1, Q2) and that women were at risk of poor mental health during and after pregnancy (Table 1: Q3, Q4, Q5). Many healthcare providers appreciated that poor maternal mental health can have negative consequences for the woman, her baby and the wider family (Table 1: Q6, Q7, Q8, Q9). Within these themes, there was an underlying willingness of the healthcare providers to provide better care. However, there were significant barriers in place.

**Table 1:** Enabling factors for the provision of maternal mental health

Sub-theme	Quote	
Awareness of mental health	Q1	“… when it comes to mental health it’s a vast variety, like it’s a whole spectrum … spectrum for poor mental health to good mental health...” (A12, KII, Doctor)
	Q2	“… some people have pre-existing … mental illnesses that carry on into pregnancy, and then you have another group … who would have the just the post-partum blues … so I think it’s a whole spectrum …” (A16, KII, Doctor)
Awareness of maternal mental health	Q3	“… most pregnant women are at risk or suffering from one or two mental conditions especially in the post-partum …” (A10, KII, Doctor)
	Q4	“We have post-partum psychological problems which is a spectrum, we have maternal blues, we have depression, we have psychosis and all postnatally …” (A4, KII, Doctor)
	Q5	“… the commonest ones we have is postpartum depression, and postpartum psychosis …” (A18, KII, Doctor)
Awareness of the effect of poor maternal mental health on the mother, baby, family	Q6	“Poor mental health can affect your day to day activities, your work, your schooling and how you relate to other persons …” (A12, KII, Doctor)
	Q7	“… if the mother is not excited or happy about giving birth to the child and there is no natural bonding between the mother and the child, the baby is neglected, the baby is not fed properly, the baby becomes malnourished …” (A3, KII, Doctor)
	Q8	“… the woman who is suffering from schizophrenia, who has paranoid delusions … can feel that they’ve sent the baby to come and harm her or something, so she will just withdraw from the baby …” (A8, KII, Doctor)
	Q9	“...if the woman is not in the best state of mind and she gives birth … the family tends to suffer a lot because they find it difficult to associate with such a person so it’s not a good thing...” (D, FGD, Junior nurse-midwife)

### Barriers

Reported challenges to the assessment of maternal mental health included a lack of time, healthcare staff shortages, staff not trained to assess and manage maternal mental health and cultural stigmas surrounding mental health. Many healthcare providers reported that a lack of time due to the large number of women attending for maternity care and the lack of staff, were challenges that made it difficult for them to screen and/or manage and/or refer women with mental health problems (Table 2: Q10, Q11, Q12).

**Table 2:** Barriers to the provision of maternal mental health

Sub-theme	Quote	
Lack of time	Q 10	“… everybody wants to deal with the more … organic things … because the outpatient department is choked, you must see as many people as you can … “(A13, KII, Doctor)
	Q 11	“But we don’t spend much time with the patient, so you don’t get to see their worries...” (A6, KII, Doctor)
	Q 12	“So, you just have so many [patients] at a time, that there’s no time to really give that kind of care that you should. We know we should be giving it, but we don’t give it, there’s really no time to do that …” (A11, KII, Doctor)
Lack of staff	Q 13	“… we need more doctors so that the workload will come down, then one can have enough time, addressing mental issue … but one doesn’t have a luxury to do it …” (A10, KII, Doctor)
	Q 14	“… everybody is stressed, even the doctor! How can a stressed person look after somebody who is stressed?” (A6, KII, Doctor)
Lack of education	Q 15	“We don’t take psychiatry serious at all … even in medical school …” (A6, KII, Doctor)
	Q 16	“I don’t think mental health has really been one of those things that is commonly taught … part of our training which is dedicated to mental health is small.” (A4, KII, Doctor)
Stigma	Q 17	“… mental health comes with some stigma” (A8, KII, Doctor)
	Q 18	“… you see we live in a society where mental health is being stigmatised … people who have mental problems are stigmatized …” (A18, KII, Doctor)
	Q 19	“… most people want to [help] but the fear of stigma wouldn’t allow them to do it” (E, FGD, Junior nurse-midwife)
	Q 20	“… you see because we live in a society where mental health is being stigmatised people who have mental problems they are being stigmatized, so, to go and discuss something like that with the person … they might think that you are tagging her as having a mental problem.” (A18, KII, Doctor)
	Q 21	“The first impression that comes to your mind when you hear mental health is hmm …. ‘this person is not in the right mind’ yeah, so it comes with its own stigma.” (A8, KII, Doctor)
Cultural beliefs	Q 22	“… they attribute it to these spiritual things, so most of the cases won’t come to the hospital … unless of course they realise, maybe, it’s getting out of hand and then they go to the pastor …” (E, FGD, Junior nurse-midwife)
	Q 23	“… [if] you’re crying more or you’re behaving differently than somebody else … they would consider it as … of spiritual origin...” (A11, KII, Doctor)
	Q 24	“the perception about schizophrenia was that people are ‘mad’ because that’s when they hallucinate, they hear auditory hallucinations and visual hallucinations they have paranoia and all that.” (A8, KII, Doctor)
	Q 25	“...in our part of the world, nobody would like to be called a ‘mad man or mad woman’, so we have to be tactful...” (A2, KII, Doctor)

Some healthcare providers commented that working in a busy teaching hospital with time pressures resulted in staff burnout and healthcare providers were keen to leave as soon as the obstetric and general physical care of the women was achieved (Table 1: Q13, Q14). Some healthcare providers commented that undergraduate and postgraduate teaching on mental health was not promoted as much as other areas of medicine (Table 1: Q15, Q16).

Many healthcare providers reported significant stigma associated with maternal mental health and that women fear being labelled as ‘not in their right mind’ if they were diagnosed with a mental disorder (Table 1: Q 17-Q21). Many healthcare providers commented that maternal mental health disorders were perceived to have ‘spiritual origins’ and that women would prefer to seek care from a religious leader as opposed to clinical care from the healthcare facility (Table 1: Q22, Q23). Some healthcare providers shared extreme views on maternal mental health and perceived women with poor mental health as ‘mad’ (Table 1: Q24, Q25).

### Solutions

Many healthcare providers were keen to discuss solutions and recommendations regarding how to introduce mental health screening as part of antenatal and postnatal care and various approaches to implementation were suggested. Healthcare providers were keen for the introduction of routine mental health guidelines and a standardised questionnaire to help guide the assessment of women as part of routine antenatal and postnatal contacts (Table 3: Q26, Q27, Q28). The use of visual posters, lectures and health talks on the topic of maternal mental health (Table 3: Q 29, Q30, Q31); education to promote awareness on the importance of mental health not only for healthcare providers, but also for women, their families and the wider community (Table 3: Q32, Q33); and a multidisciplinary team approach, collaborating with psychologists and/or psychiatrists in an additional clinic, as part of routine maternity clinics, (Table 3: Q34, Q35, Q36) were suggested to encourage a more comprehensive and holistic approach to maternal mental healthcare.

**Table 3:** Suggested solutions for the provision of a maternal mental health service

Sub-theme	Quote	
Guidelines	Q 26	“I think there should be guidelines …” (A4, KII, Doctor)
	Q 27	“There should be guidelines so that there’s a kind of purpose, there’s a reason why you are doing it, so you can’t just be screening women with no purpose or no guidelines.” (A8, KII, Doctor)
	Q 28	“I just wish it becomes part of the routine screening of our patients because if you are able to detect … those with such conditions or who need help early that will prevent us from getting to them late” (A20, KII, Doctor)
	Q 29	“if it is a poster that I turn, and I look, and I see something that reminding me that ‘we must ask this’ it’s helpful … something so people will be reminded everywhere in your hospital ‘mental health in women is important” (A6, KII, Doctor)
Antenatal screening	Q 30	“We could include it into our antenatal book then routinely, and postnatal of course, routinely when the patient comes as part of the questions we ask …” (A12, KII, Doctor)
	Q 31	“… at the first visit, the booking visit, if we had … a structured questionnaire or a tool that could be used, it will go a long way so that right from booking...potential problems are picked up early enough...” (A16, KII, Doctor)
Education for mothers	Q 32	“It should be part of...the antenatal clinic, because when they come we give them talks …” (A20, KII, Doctor)
Education of healthcare providers	Q 33	“… we need to have public education, we need to educate people, we need to educate ourselves as healthcare providers, we need to educate the pregnant women, we need to come up with policies, we need to improve and set up a structured plan in a way to attack this situation …” (A3, KII, Doctor)
		“… we need to train [healthcare providers] we have to get the techniques to do it … and you need to get things in place to do it...” (A16, KII, Doctor)
Multidisciplinary team approach	Q 34	“I would say that there should be separate unit in the antenatal clinic where a psychiatrist or psychiatric nurse or psychologist could do the screening and counselling (A1, KII, Doctor)
	Q 35	“… having a multi-disciplinary approach to it involving the physicians coming to [the antenatal clinic] some days … if they are there at the same clinic it might improve the management than losing and managing them …” (A9, KII, Doctor)
	Q 36	“I think we need psychiatry units … so that we know where we are directing our patients to when it comes to mental health” (C, FGD, Junior nurse-midwife)

## Discussion

### Statement of principal findings

Many healthcare providers were aware of the problem and impact of poor maternal mental health and would be keen to support women. Healthcare providers understand that maternal mental health should be continuously assessed along the continuum of care from early pregnancy to the late postnatal period, as early identification would enable early referral and treatment (depending on availability). However, healthcare providers do not currently screen women for maternal mental health problems during and after pregnancy due to a lack of training and/or time and because mental health is often considered a spiritual issue within the cultural setting. Further education and training of healthcare providers (undergraduate and postgraduate) would be useful to develop their confidence to approach this potentially culturally sensitive topic and to enable routine screening and management of maternal mental health disorders during and after pregnancy. Healthcare providers need support from a wider multidisciplinary team, ideally with a psychiatrist to work together in a combined approach to ensure women receive the best possible care in a more holistic and comprehensive way. The healthcare providers interviewed suggested that education and sensitisation programmes regarding the aetiology and evidence-based management for mental health during and after pregnancy that would be aimed at the community, families and women would be beneficial. The healthcare providers recommended the introduction of culturally appropriate routine mental health screening tools for use within existing services.

### Strengths of the study

This study assessed the knowledge and attitude of healthcare providers regarding maternal mental health during and after pregnancy and, to our knowledge is the first study to assess this subject in a low resource setting. This study discusses solutions that can support future policy and programme development to introduce and establish routine maternal screening for mental health during and after pregnancy in a low resource setting. A wide spectrum of responses were obtained by interviewing different cadres of healthcare providers (both female and male), who worked in different departments within the maternity setting and had varied level of experiences. Most of the healthcare providers interviewed welcomed the discussion surrounding screening for maternal health in pregnancy, recognised it as a neglected area and were keen to contribute to solutions in their settings.

### Limitations of the study

This study population comprised mainly doctors providing routine maternity care in a large teaching hospital and excludes other cadres of healthcare providers who do not provide maternity care and may have alternative perspectives or different insights. Similarly, this study was carried out in an urban setting and the findings cannot be assumed to be the same in other settings. There is a need to assess the views of community-based healthcare providers in different settings who may have different cultural perceptions, beliefs and experience. Their opinions would be important to ensure a seamless home to hospital continuum of care regarding maternal mental health referral and treatment pathways.

### How does this study relate to other literature?

In our study, healthcare providers felt unable to routinely screen women for mental health due to the lack of training, lack of resources and lack of time in a busy urban healthcare facility. Furthermore, healthcare providers reported that mental health was associated with significant stigma. These findings are similar to those from other studies from a variety of settings where stigma and discrimination against women with mental health disorders still exist [19]. In our study, mental health was surrounded by a shroud of cultural misconception, with a reported cultural context of mental illness being associated with extreme cases such as ‘those mad homeless people, living on the streets, naked and in poverty’. This understanding is similar to other studies in low resource settings, where the term ‘madness’ was used to describe mental health, and people with mental illness being perceived as ‘dishevelled and homeless’ [20]. In our study, many healthcare providers explained that, culturally, mental disorders were believed to have ‘spiritual origins’. Similar responses were found in another study in Ghana where mental ill-health was associated with spiritual influences and methods of treatment included visits to a religious leader or harsh beatings to remove ‘evil spirits’ [20]. In many LMIC settings, families tend to seek care from traditional healers and/or churches as opposed to seeking clinical care at healthcare facilities [21]. These perceptions and beliefs are ingrained in the cultural context making it difficult for healthcare providers to discuss mental health as part of a routine ‘normal’ health consultation with a woman during and/or after pregnancy. Lack of education and awareness surrounding the spectrum and available management for mental illness within the communities may continue to contribute to misconceptions on mental health [21].

In our study, many healthcare providers suggested that an integration of routine mental health guidelines into the existing maternal health system would be beneficial. There are many international clinical and policy guidelines on who should enquire, screen and manage maternal mental health during and after pregnancy and how this should be conducted, including identification, counselling, documentation, first line medication and provision of higher referral pathways [8, 15]. Furthermore, in high income countries, there are recommendations that every woman should be asked about her emotions every time she is seen by a healthcare professional during and after pregnancy, in an open and non-judgemental way, highlighting that screening tools may help, but a prompt to ask is important also [14]. However, the practicalities associated with the implementation and acceptability of these recommendations in countries such as Ghana is currently uncertain. There is a need to further understand how maternal mental health is experienced and understood within different cultural contexts to ensure that the interventions to be implemented are culturally and contextually appropriate [1]. There is also debate as to who is most suitable to enquire, screen for and manage maternal mental health and at what level of the health system (community, primary or secondary health care level) across LMIC. In many HIC, specially trained midwives routinely assess, support and provide further referral between different levels of care [14]. This approach in a low resource setting requires further research. A systematic review in 2013 assessed the effectiveness of interventions to improve the maternal mental health in LMIC and concluded that interventions could be delivered by supervised non-specialists [17]. An intervention package for perinatal depression (cognitive behaviour therapy, psycho-education, problem solving, parenting skills) is being delivered by community midwives with support from doctors in facilities and enhanced compliance with mobile phones in Nigeria [22]. Evaluation is awaited.

## Conclusion

Across many LMIC, women are increasingly accessing antenatal care and there is now a window of opportunity to adapt and amend available care packages to include routine screening and management of maternal mental health during and after pregnancy as a component of comprehensive quality of care. Many healthcare providers are keen to help women with mental illness during and after pregnancy if resources are made available and if a culturally appropriate approach is used. This study highlights the need to understand the complexity of factors associated with maternal mental health and provides recommendations to develop screening approaches in low resource settings. Clear effective referral pathways and support for women who report mental health concerns during and after pregnancy would be beneficial and require further research as to how best provide this care in low resource settings.

## Data Availability

The dataset used and analysed during the current study are available from the corresponding author on reasonable request.

## Abbreviations

ANC: Antenatal care
FGD: Focus group discussions
HIC: High income country
KII: Key informant interviews
LMIC: Low and middle-income countries
LSTM: Liverpool School of Tropical Medicine
PNC: Postnatal Care
UK: United Kingdom
WHO: World Health Organization

## References

[1] Kathree T, Selohilwe OM, Bhana A, Petersen I (2014). Perceptions of postnatal depression and health care needs in a south African sample: the “mental” in maternal health care. BMC Womens Health.

[2] Miller S, Abalos E, Chamillard M, Ciapponi A, Colaci D (2016). Comandé et al. beyond too little, too late and too much, too soon: a pathway towards evidence-based, respectful maternity care worldwide. Lancet..

[3] World Health Organization (1946). Constitution of the World Health Organization as adapted by the international health conference.

[4] United Nations. Every Woman, Every Child: Global Strategy; 2015. Available from: http://www.everywomaneverychild.org/global-strategy-2.

[5] United Nations (2015). Transforming our world: the 2030 agenda for sustainable development.

[6] World Health Organisation. Maternal Mental Health; 2017. Available at: http://www.who.int/mental_health/maternal-child/maternal_mental_health/en/.

[7] Vos T, Barber RM, Bell B, Bertozzi-Villa A, Biryukov S, Bollger I et al. Global, regional, and national incidence, prevalence, and years lived with disability for 301 acute and chronic diseases and injuries in 188 countries, 1990–2013: a systematic analysis for the Global Burden of Disease Study 2013. Lancet. 2015;386(9995):743–800. 10.1016/S0140-6736(15)60692-4 PMC4561509 26063472

[8] Fisher J, de Mello MC, Patel V, Rahman A, Tran T, Holton S (2012). Prevalence and determinants of common perinatal mental disorders in women in low-and lower-middle-income countries: a systematic review. Bull World Health Organ.

[9] Gelaye B, Rondon MB, Araya R, Williams MA (2016). Epidemiology of maternal depression, risk factors, and child outcomes in low-income and middle-income countries. Lancet Psychiatry.

[10] Leigh B, Milgrom J (2008). Risk factors for antenatal depression, postnatal depression and parenting stress. BMC Psychiatry.

[11] Satyanarayana VA, Lukose A, Srinivasan K (2011). Maternal mental health in pregnancy and child behavior. Indian J Psychiatry.

[12] Manikkam L, Burns JK (2012). Antenatal depression and its risk factors: an urban prevalence study in KwaZulu-Natal. S Afr Med J.

[13] Patel V, Rahman A, Jacob K, Hughes M (2004). Effect of maternal mental health on infant growth in low income countries: new evidence from South Asia. Br Med J.

[14] National Institute for Health and Care Excellence. Antenatal and postnatal mental health: clinical management and service guidance: Postnatal Care; 2015. Available at: https://www.nice.org.uk/guidance/cg192 31990493

[15] Mental Health. Ghana, Situational analysis: a country report, WHO 2007. Available from:https://www.who.int/mental_health/policy/country/GhanaCoutrySummary_Oct2007.pdf?ua=1

[16] Rahman A, Fisher J, Bower P, Luchters S, Tran T, Yasamy MT (2013). Interventions for common perinatal mental disorders in women in low-and middle-income countries: a systematic review and meta-analysis. Bull World Health Organ.

[17] World Health Organization. World Health Statistics 2017. Geneva: World Health Organization; 2017. Available from:http://www.who.int/gho/publications/world_health_statistics/2017/en/.

[18] Braun V, Clarke V (2006). Using thematic analysis in psychology. Qual Res Psychol.

[19] Ngui EM, Khasakhala L, Ndetei D, Roberts LW (2010). Mental disorders, health inequalities and ethics: a global perspective. Int Rev Psychiatry.

[20] Read UM, Adiibokah E, Nyame S (2009). Local suffering and the global discourse of mental health and human rights: an ethnographic study of responses to mental illness in rural Ghana. Glob Health.

[21] Asamoah MK, Osafo J, Agyapong I (2014). The role of Pentecostal clergy in mental health-care delivery in Ghana. Ment Health Religion Cult.

[22] Gureje O, Oladeji BD, Araya R, Montgomery AA, Kola L, Kirmayer L (2015). Expanding care for perinatal women with depression (EXPONATE): study protocol for a randomized controlled trial of an intervention package for perinatal depression in primary care. BMC Psychiatry..

