# Correction to: “I just wish it becomes part of routine care”: healthcare providers’ knowledge, attitudes and perceptions of screening for maternal mental health during and after pregnancy: a qualitative study

**DOI:** 10.1186/s12888-019-2295-0

**Published:** 2019-10-18

**Authors:** Mary McCauley, Abigail Brown, Bernice Ofosu, Nynke van den Broek

**Affiliations:** 1 Centre for Maternal and Newborn Health, Liverpool School of Tropical Medicine, Pembroke Place, Liverpool, L3 5QA UK; 2 Korle-Bu Teaching Hospital, Accra, Ghana

**Correction to: BMC Psychiatry (2019) 19:279**

**https://doi.org/10.1186/s12888-019-2261-x**

Following publication of the original article [1], we have been notified of a few mistakes in the display of the author names. The publisher apologizes for the inconvenience.

In this Correction the incorrect and correct author names are shown:

- Mary Mccauley should read Mary McCauley
- Brown Abigail should read Abigail Brown
- Ofosu Bernice should read Bernice Ofosu
- Nynke Van Den Broek should read Nynke van den Broek

The original article has been corrected.
